# A Magnetic Flux Leakage Detector for Ferromagnetic Pipeline Welds with a Magnetization Direction Perpendicular to the Direction of Travel

**DOI:** 10.3390/s24165158

**Published:** 2024-08-10

**Authors:** Wei Cui, Zhongmin Xiao, Ziming Feng, Jie Yang, Qiang Zhang

**Affiliations:** 1 School of Energy and Power Engineering, University of Shanghai for Science and Technology, Shanghai 200093, China; cuiwei@usst.edu.cn (W.C.); yangjie@usst.edu.cn (J.Y.); 2 School of Mechanical and Aerospace Engineering, Nanyang Technological University, 50 Nanyang Avenue, Singapore 639798, Singapore; 3 School of Mechanical and Electrical Engineering, Wenzhou University, Wenzhou 325035, China; 4 School of Mechanical Science and Engineering, Northeast Petroleum University, Daqing 163318, China; tical2012@163.com

**Keywords:** magnetic flux leakage testing, ferromagnetic pipelines, testing instruments, pipeline welds, defect quantification method

## Abstract

For the sake of realizing the safety detection of natural gas and petroleum pipeline welds, this paper designs a ferromagnetic pipeline weld magnetic flux leakage detector based on the calculation of the magnetic circuit of the detection probe, with the magnetization direction perpendicular to the traveling direction. The traditional pipeline magnetic flux leakage detection device uses a detection system mode in which the magnetization direction is parallel to the direction of travel. However, due to the structural characteristics of the weld, the traditional detection system mode is not applicable. Since the weld magnetic flux leakage detector needs to travel along the direction of the weld, the detector designed in this paper rotates the magnetizer 90 degrees along the direction of the weld seam so that the magnetization direction is perpendicular to the direction of travel, breaking through the technical barrier that make traditional magnetic flux leakage detection devices unsuitable for weld detection. The detection device includes a magnetizing structure, a data sampling device, and a driving and traveling device. The magnetic flux leakage signal collected by the detector is converted into a digital image in the form of a grayscale matrix. Using mathematical morphology and chain code algorithms in image processing technology, a pipeline weld defect inversion software system is developed, and a preliminary quantitative analysis of pipeline weld defects is achieved. The application of this technology enables the inspection and protection of oil and gas pipeline welds throughout their life cycle, broadens the scope of existing inspection objects, and is of great safety significance for ensuring national public security.

## 1. Introduction

Natural gas and petroleum are classified as dangerous goods under key national supervision, and long-distance pipelines used to transport gas and petroleum resources may pose significant sources of danger. Therefore, the inspection and protection of natural gas and petroleum pipelines are major issues involving the prevention and control of national safety production accidents. In long-distance pipeline projects, pipeline welds are the weak links. If defects such as cracks appear in the joints, significant safety risks will be hidden in the operation of the pipeline [1,2,3,4,5,6,7,8,9]. Therefore, accurate and rapid detection of pipeline welds has great safety significance and economic value for ensuring national public security. Magnetic flux leakage detection technology has the advantages of easy automation, excellent detection reliability, and superior detection efficiency. Therefore, it is widely used in the detection of tank floors, tank walls in tank areas, and pipelines. Since the detection system mode of the traditional pipeline magnetic flux leakage detection device in which the magnetization direction is parallel to the direction of travel is not suitable for weld detection [10,11,12,13,14], in order to enable the detection device to advance along the direction of the pipeline weld, this paper rotates the magnetization direction by 90 degrees so that the magnetization direction is perpendicular to the direction of travel. The detection device collects the magnetic flux leakage signal generated by the contour of the weld itself and the magnetic flux leakage signal generated by the defects. The two overlap, resulting in signal complexity and posing challenges to the quantification of pipeline weld defects.

The quantification of defects detected by magnetic flux leakage technology mainly focuses on the analysis of the magnetic flux leakage signal inversion algorithm. Joshi applied an RBF neural network to the inversion of magnetic flux leakage signals and achieved good inversion results [15]. Priewald et al. reconstructed complex-shaped defects based on finite element simulation, and the results showed that the algorithm has high accuracy [16]. Wang, Jiang, and others applied the neural network pattern recognition method to defect magnetic flux leakage detection and proposed a method for the quantitative identification of defects using a BP network and a wavelet neural network, realizing the automatic extraction of defect leakage magnetic field characteristics and the intelligent recognition of defect outline dimensions [17,18]. Carvalho and others used neural networks to classify pipeline weld defect signals, including external corrosion, internal corrosion, and lack of penetration. They also performed preprocessing such as Fourier analysis on the signals, thus improving the recognition performance of the neural network in classifying defects [19]. Geng and others used the vgg16 network to classify and predict pipeline weld defects based on snapshots of magnetic flux leakage detection signals, with an accuracy of 78% [20]. The above research work has achieved quantitative inversion of a single magnetic flux leakage signal (for example, quantitative inversion of pipeline defects) and qualitative inversion of pipeline welds but has not yet addressed the quantitative inversion of pipeline welds; this is because when detecting pipeline welds using the magnetic flux leakage method, the magnetic flux leakage signals generated by the contour of the weld itself overlap with the magnetic flux leakage signals generated by the defects. Therefore, this paper proposes using image processing technology in signal processing. The basic principle is to obtain the defect area through image segmentation and image recognition of the collected defect images. On this basis, the geometric characteristics, gray value characteristics, texture characteristics, etc., of the defects are further obtained. At present, image processing technology has been successfully used in weld radiographic inspection and weld ultrasonic inspection systems. For example, Shao et al. used the automatic detection of weld defects from digital images of double-sided weld X-ray films [21]. Gao and others introduced compressed sensing theory into X-ray weld image defect judgment [22]. Hu et al. studied a new method for weld defect identification based on the fusion of ultrasonic signals and images, respectively extracting the ultrasonic echo signal characteristics of defects and the morphological characteristics of defect images. This method realizes the identification of multiple types of defects [23]. However, the introduction of image processing technology into the weld magnetic flux leakage detection method is still in its infancy. This paper explores a pipeline weld defect quantification method based on image recognition, proposes a quantitative research method for pipeline weld defects based on the integration of mathematical morphology and chain code algorithms, develops a pipeline weld defect inversion software system, and realizes a preliminary quantitative study on pipeline weld defects.

## 2. Magnetization Structure Design

### 2.1. Detection Probe Magnetic Circuit Design

The magnetizing structure dimensions are designed based on the following pipeline specifications: *ϕ*1219 mm × 18.4 mm, material X80, weld reinforcement 2 mm, and weld width 22 mm. The permanent magnet in the magnetized structure is made of rubidium–iron–boron (Nd-Fe-B) N48, and the pole shoes and armature are made of industrial pure iron, a soft magnetic material with good magnetic permeability. In order to adapt to the curvature of the pipeline, arc pole shoes are designed to ensure that the distance between the pole shoes and the oil and gas pipeline is relatively uniform. The magnetizing structure, composed of permanent magnets, pole shoes, and armatures, together with the air gap and the oil and gas pipeline and welds being tested, form a magnetic circuit. It can be seen from the demagnetization curve of N48 [24] that at 20 °C, *B_d_* = 7.02 KGs, *H_d_* = 6.76 Koe, *B_m_* = 6.9 KGs, *H_m_* = 6.8 KOe, and the magnetic energy product *BH_m_* = 46.92 MGsOe.

**Figure 1:**
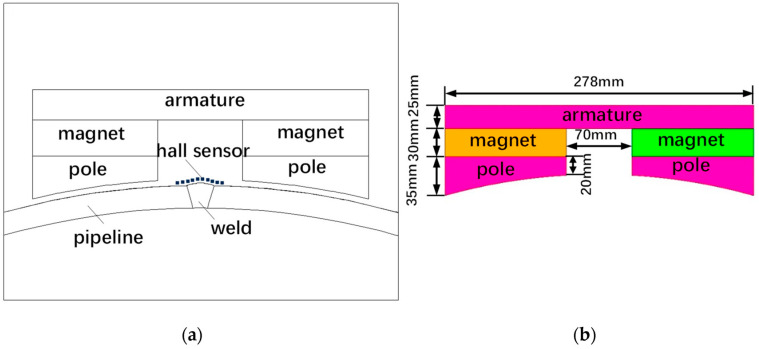
Schematic diagram of the structure and dimensions of the magnetic flux leakage probe: (**a**) Magnetic flux leakage probe structure; (**b**) Magnetized structure dimensions.

The basic steps of magnetic circuit design include first determining the approximate structure and size of each part of the magnetic circuit, material type, and performance, and then solving the magnetic field and calculating the magnetic flux distribution in space. If the results are far from the requirements, the original size and performance are optimized until the calculated results are basically consistent with the design requirements. The schematic diagram of the detection probe is shown in Figure 1a. The magnetic circuit analysis and calculations are performed based on this structure. After determining the structural size and magnetic source of the magnetic circuit, various methods can be used to analyze and calculate the magnetic characteristics of each part of the magnetic circuit, such as the magnetic permeability method, the magnetic charge integration method, the boundary element method, and the finite element method. Among them, the magnetic permeability method uses the calculated magnetic permeance of each part of the magnetic circuit to calculate the air gap magnetic flux density [25]. This paper uses the magnetic permeability method to calculate the excitation magnetic circuit of the pipeline weld. This method is relatively simple to analyze and calculate. It can directly obtain the magnetic field characteristic parameters, and the calculation amount is small, and the results are relatively reliable.

The magnetic circuit design steps [25] are as follows:

(1) Select the magnetic circuit structure and permanent magnet working point according to the design requirements (value of the magnetic induction intensity of the air gap, *B_g_*; value of the air gap area, *A_g_*; value of the air gap length, *L_g_*). When selecting the magnetic circuit structure, the size of the magnet needs to be considered in conjunction with the performance of the permanent magnet, and the position of the magnet should be as close to the air gap as possible. The size of the pole shoes should be large enough so that the magnetic flux passing through it does not saturate the pole shoes; that is,

(1) $$\emptyset =B_{j}\cdot A_{j}$$

where ∅ is the magnetic flux, *B_j_* is the magnetic induction intensity corresponding to the maximum permeability, and *A_j_* is the area corresponding to the maximum permeability.

If *B_j_* is equal to the saturation magnetic flux density, the magnetic resistance of the pole shoes itself increases a lot, and the magnetic potential drop increases; that is, the magnetomotive force loss is too large.

(2) Estimate the magnetic leakage coefficient *k_f_* and the magnetoresistive coefficient *k_r_*, and use Equations (2) and (3) to preliminarily calculate the magnet size at the operating point, including the permanent magnet area *A_m_* and the permanent magnet length *L_m_*.

(2) $$\emptyset =B_{m}A_{m}=k_{f}B_{g}A_{g}$$

where *B_m_* is the magnetic induction intensity at the operating point, *A_m_* is the permanent magnet area at the operating point, *B_g_* is the magnetic induction intensity of the air gap, and *A_g_* is the air gap area.

(3) $$F={-H}_{m}L_{m}=k_{r}H_{g}L_{g}$$

where *F* is the magnetomotive force, *H_m_* is the magnetic field intensity at the operating point, *L_m_* is the permanent magnet length at the operating point, *H_g_* is the magnetic field intensity of the air gap, and *L_g_* is the air gap length.

From Formulas (2) and (3), the equation below can be obtained:

(4) $$B_{g}^{2}={(-H}_{m}B_{m})\frac{V_{m}}{V_{g}}\cdot \frac{\mu_{0}}{k_{f}k_{r}}$$

where *B_g_* is the magnetic induction intensity of the air gap, *V_m_* is the permanent magnet volume, *V_g_* is the air gap volume, and $\mu_{0}$ is the vacuum permeability.

(3) Based on the sizes of the permanent magnet, pole shoes, and armature, calculate the total magnetic permeance *G* of the entire magnetic circuit. In addition, the magnetic leakage coefficient *k_f_* needs to be calculated.

(4) Substitute the calculated total magnetic permeance *G* and magnetic leakage coefficient *k_f_* as well as the internal resistance *r* of the magnet and the pole magnetic resistance *R*, plus the original operating point of the magnet (*B_d_*, *H_d_*), into the following Formula (5):

(5) $$B_{g}=\frac{F}{k_{f}A_{g}(r+R+\frac{1}{G})}$$

where *r* is the internal resistance, *R* is the pole magnetic resistance, and *G* is the total magnetic permeance of the entire magnetic circuit.

Check whether the obtained *B_g_* value meets the requirements. If it does not meet the requirements, start over again until it meets the design requirements.

Through the above calculation process, *k_f_* is calculated in an iterative manner, and the size of the magnetized structure is obtained, as shown in Figure 1b.

### 2.2. Numerical Simulation of the Magnetized Structure

**Figure 2:**
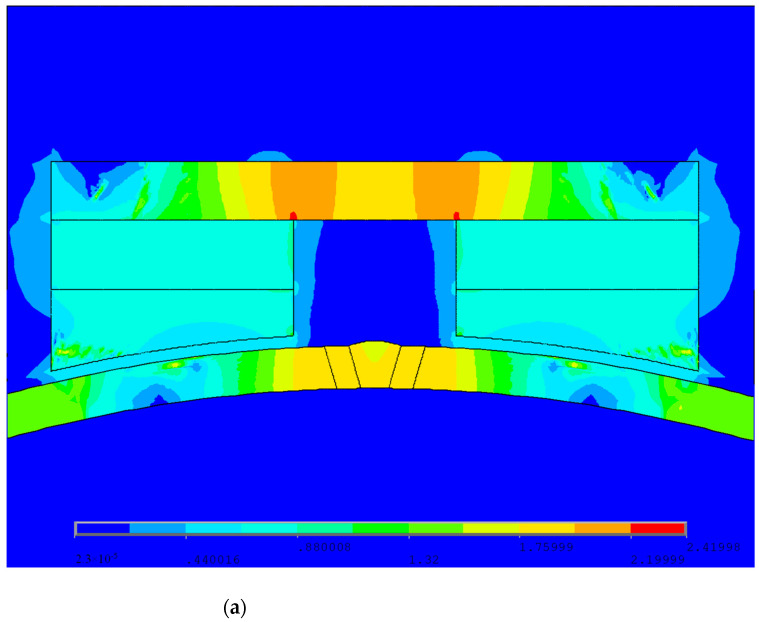

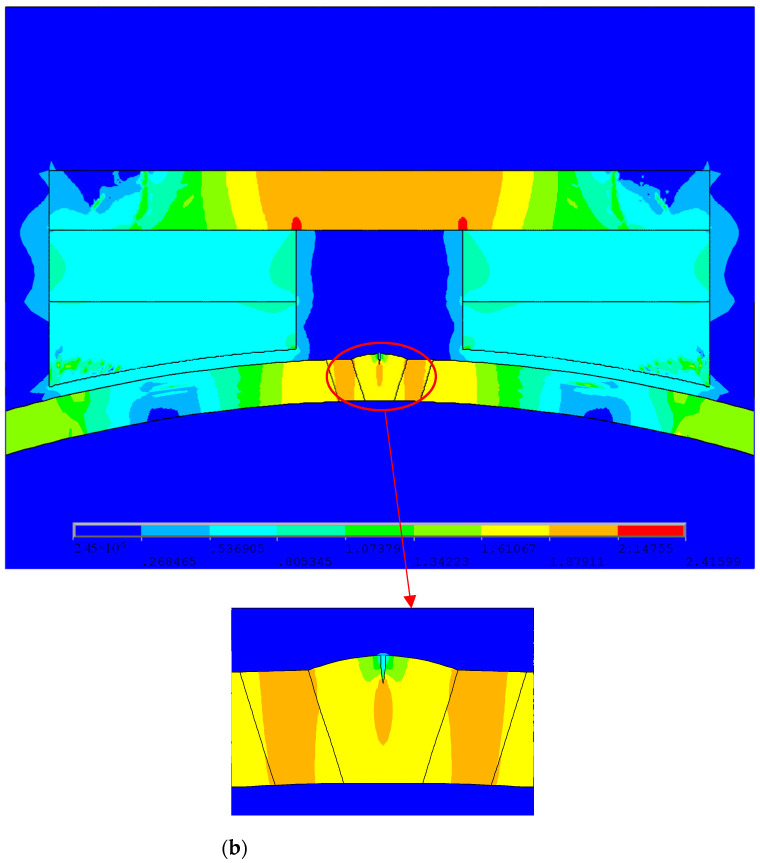

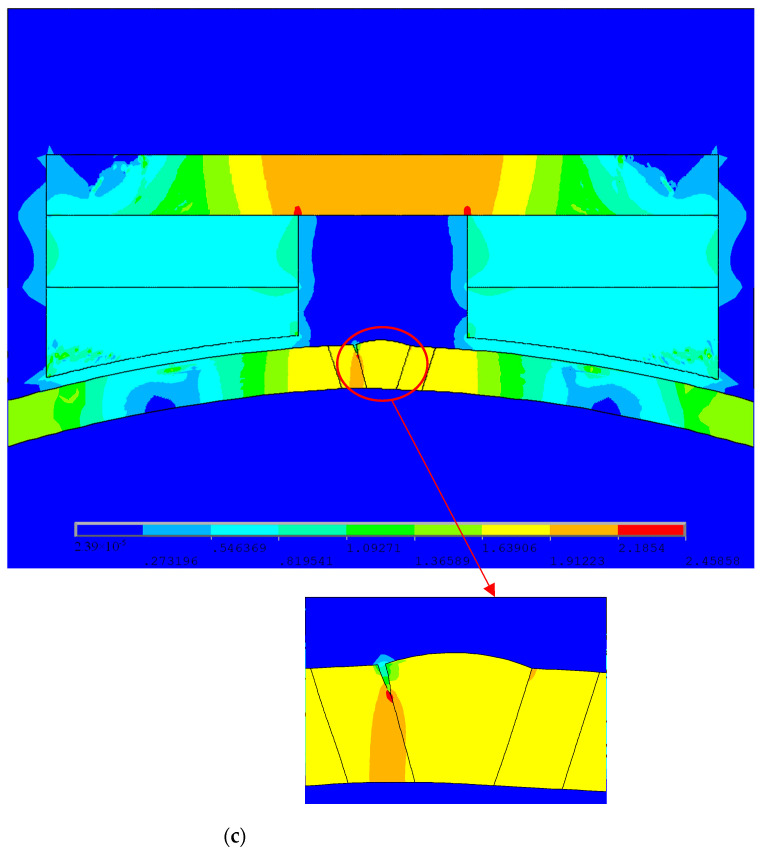
Magnetic induction intensity cloud charts showing (**a**) a defect-free X80 pipeline weld, (**b**) cracks in the weld bead of the X80 pipeline, and (**c**) cracks in the fusion zone of the X80 pipeline weld.

The magnetic circuit design requires that the magnetic field intensity of the magnetic circuit should make the pipeline reach saturation or a near-saturation magnetization state, and the volume of the magnetic circuit structure should be as small as possible. The specific requirement for the magnetization intensity is greater than 80% of the saturation magnetization intensity. Pipelines are usually made of low-carbon steel material with a saturation magnetic induction intensity of 1.6 T, so the magnetization intensity detected must reach 1.2 T or above. Figure 2a–c show the magnetic induction intensity cloud maps of the X80 pipeline weld without defects, cracks on the X80 pipeline weld, and cracks in the fusion zone of the X80 pipeline weld, respectively. The magnetic flux leakage detector for the ferromagnetic pipeline welds designed in this paper meets the requirement that the magnetic induction intensity at the position of the pipeline weld be greater than 1.2 T.

## 3. Development of Continuous Non-Contact Magnetic Flux Leakage Scanning Technology for Pipeline Welds

### 3.1. Design of a Continuous Non-Contact Magnetic Flux Leakage Scanner for Pipeline Welds

**Figure 3:**
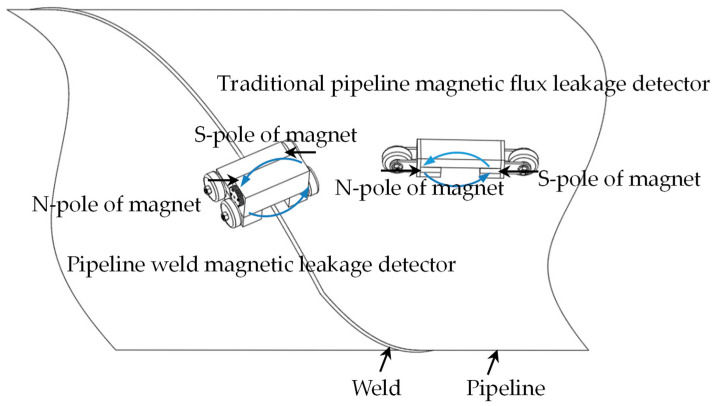
Comparison diagram of the magnetization structure.

Traditional pipeline magnetic flux leakage detectors use a detection system mode in which the magnetization direction is parallel to the direction of travel. However, due to the structural characteristics of the weld, the traditional detection system mode is not applicable. Since the weld magnetic flux leakage detector needs to travel along the direction of the weld, the detector designed in this paper rotates the magnetizer 90 degrees along the direction of the weld seam so that the magnetization direction is perpendicular to the direction of travel, breaking through the technical barrier of traditional magnetic flux leakage detection devices not being suitable for weld detection. The magnetization structure comparison diagram of the pipeline weld magnetic flux leakage detection mode designed in this paper is different from the traditional pipeline magnetic flux leakage detection mode, as shown in Figure 3.

The magnetizing mechanism includes the armature, permanent magnet, and pole shoes. The excitation circuit consists of an armature, a permanent magnet, pole shoes, a magnetizing air gap, and a weld of the pipeline being tested. The permanent magnet uses high-performance rubidium–iron–boron. The permanent magnet is the excitation source of the magnetizing structure and provides magnetic flux for the magnetic circuit. The function of the pole shoes is to protect the permanent magnet, prevent damage to the magnet due to collision and friction, and, at the same time, increase the uniformity of the magnetic field. The armature plays a role in conducting magnetism in the magnetic circuit. It connects various parts of the magnetic circuit in series to form a magnetic circuit. The armature is generally made of soft magnetic materials, which can reduce magnetic resistance. In addition, the armature also plays the role of structural support. Multiple threaded holes are opened on the armature to fix and install other ancillary structures, such as guard plates.

**Figure 4:**
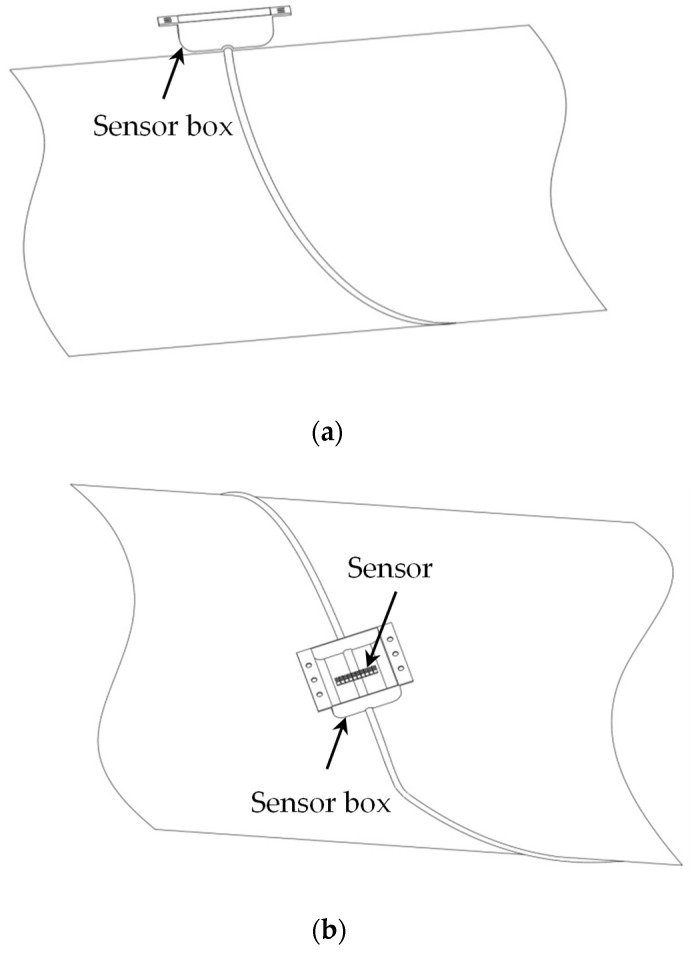
Sensor diagram: (**a**) Schematic diagram of the sensor box shape matching the welding seam; (**b**) Schematic diagram of the Hall sensor array in the sensor box.

Compared with the traditional pipeline magnetic leakage detector, the sensor arrangement of the pipeline weld magnetic leakage detector has also been changed. The sensors are arranged inside the sensor box parallel to the bottom surface of the pole shoes, so the sensor array direction is parallel to the magnetization direction and perpendicular to the direction of travel. These sensors pick up the leakage magnetic field produced by the defect and convert the magnetic signal into an electrical signal. This detector uses Hall sensors, which are packaged in a stainless-steel sensor box. The Hall sensors of the SS459A model are selected in this paper. This detector uses 10 sensors to meet the detection requirements and can detect magnetic flux changes in the order of 10^−3^ T. The schematic diagram of the Hall sensor array in the sensor box is shown in Figure 4a. Due to the presence of weld reinforcement, the sensor box is designed to match the contour of the weld. On the one hand, this design reduces the distance between the sensors and the pipeline weld, and on the other hand, it ensures that the lift-off value between the sensors and the pipeline and the weld is equal. The schematic diagram of the matching of the shape of the sensor box and the weld is shown in Figure 4b.

**Figure 5:**
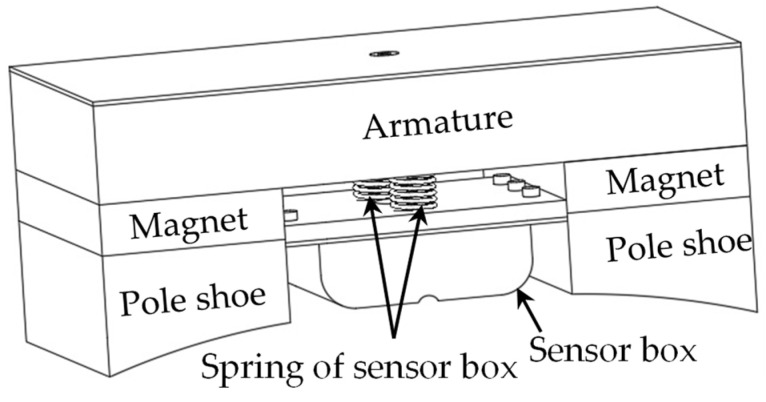
Schematic diagram of the sensor box location.

The sensor box is joined via threaded connections to the armature through two height-adjustment screws and fixed between the two magnetic poles under the armature to place and protect the Hall sensors. The spring is squeezed by the lower end surface of the armature and the upper end surface of the sensor box and generates a certain elastic force, which can make the sensor box closer to the pipeline weld under a certain lift-off value requirement. The sensor box can use the spring to buffer the impact and avoid deformation or damage of the sensor box. The schematic diagram of the sensor box location is shown in Figure 5.

### 3.2. Working Principle of the Continuous Non-Contact Magnetic Flux Leakage Scanner for Pipeline Welds

**Figure 6:**
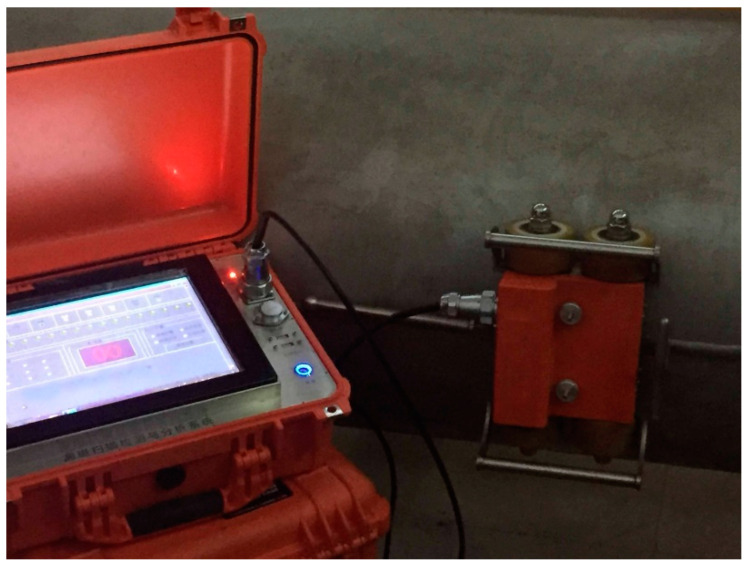
Continuous non-contact magnetic flux leakage scanner for pipeline welds.

Place the detector on the outer wall of the pipeline weld to be inspected, as shown in Figure 6. Adjust the sensor height adjustment screw to make the bottom surface of the sensor box fit closely with the pipeline weld. Turn on the system power and industrial computer and enter the detection system interface. Push the driving handle to make the detector move straight along the pipeline weld. The magnetizing structure of the detector passes through the magnetized pipeline weld area between the two pole shoes, making the pipeline weld in this section close to magnetic saturation. When defects exist, magnetic field lines can twist and spill. The leaked magnetic signal is captured by the Hall sensors located inside the sensor box. The data sampling device consists of the Hall sensors and corresponding signal amplification circuits, rotating photoelectric pulse encoders, and data sampling cards. Among them, the gear of the photoelectric pulse encoder is connected with any traveling wheel in the detector. Every time the photoelectric pulse encoder rotates at a certain angle, an electrical pulse signal will be generated, which will be transmitted to the corresponding signal-receiving end of the data sampling card, and the data sampling card will convert it into corresponding mileage data. The encoder used in this paper is the E6A2 rotary photoelectric pulse encoder, and the sampling spacing used is 2 mm. At the same time, the magnetic induction element (the Hall sensors) converts the detected magnetic flux leakage signal into an electrical signal, which is amplified by the signal amplifier circuit and then transmitted to the corresponding signal-receiving end of the data sampling card. The data sampling card then converts it into the corresponding magnetic flux leakage signal data. Thereafter, the data sampling card transmits the mileage data and magnetic flux leakage data to the industrial computer pre-installed with magnetic flux leakage signal analysis software. This process completes the defect scanning work.

Driving and traveling devices include driving handles, fixed-distance wheels, traveling wheels, etc. The detector is equipped with traveling wheels on both sides, which can move forward in a straight line at a constant speed along the pipeline in the manual driving mode. Guard plates are installed around and on the upper side of the detector. The guard plates protect the magnetization device and prevent damage to the device from external collisions. The magnetizing device is equipped with a handle and a driving handle along the direction of travel. The handle is used to pick up, place, and transport the detector. The driving handle pushes or pulls the detector to drive the detector forward along the direction of the pipeline weld. Driven by the fixed-distance wheel, the photoelectric pulse encoder counts the number of revolutions of the wheel, converts it into corresponding mileage data, and transmits it to the industrial computer. This process completes the positioning of defects. Finally, the pipeline weld defect inversion software developed for this project, which was prefabricated on the industrial computer, completes the qualitative identification and quantitative analysis of defects and generates a detection report.

This detector is powered by a DC power supply, and the collected signal waveforms can be displayed on a touch screen industrial computer in real time, and the data can be saved. After the pipeline weld detection is completed, the data need to be stored. The system power is turned off, and the detector is removed from the pipeline weld by driving the handle and placed on a clean, ferromagnetic material-free platform, which can then be packed or transported.

## 4. Experimental Study on the Magnetic Flux Leakage Detection of Pipeline Welds

**Figure 7:**
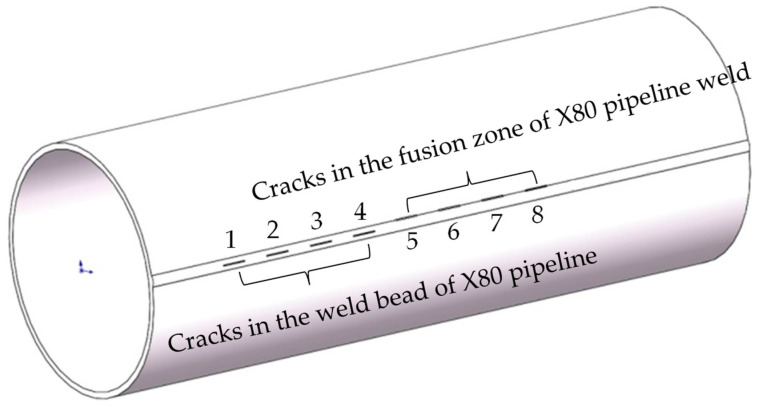
Schematic diagram of prefabricated defects in experimental pipelines.

**Figure 8:**
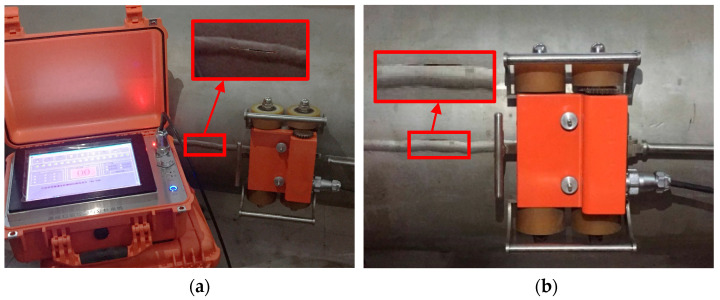
Experimental study on the magnetic flux leakage detection of pipeline welds: (**a**) Cracks in the weld bead of the X80 pipeline; (**b**) Cracks in the fusion zone of the X80 pipeline weld.

**Figure 9:**
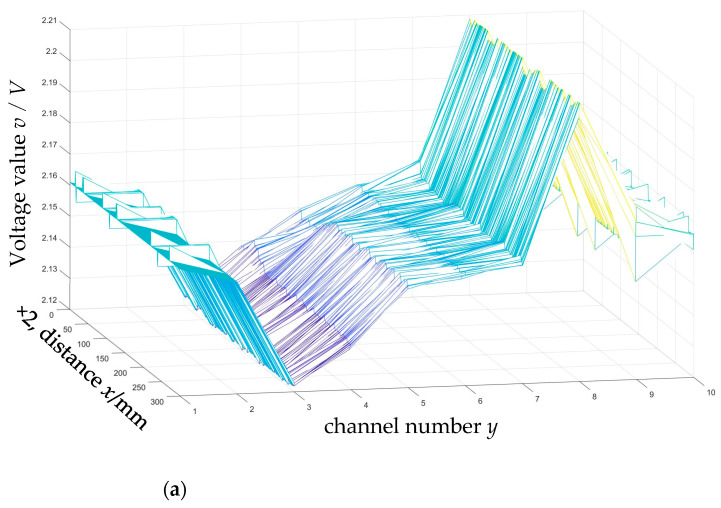

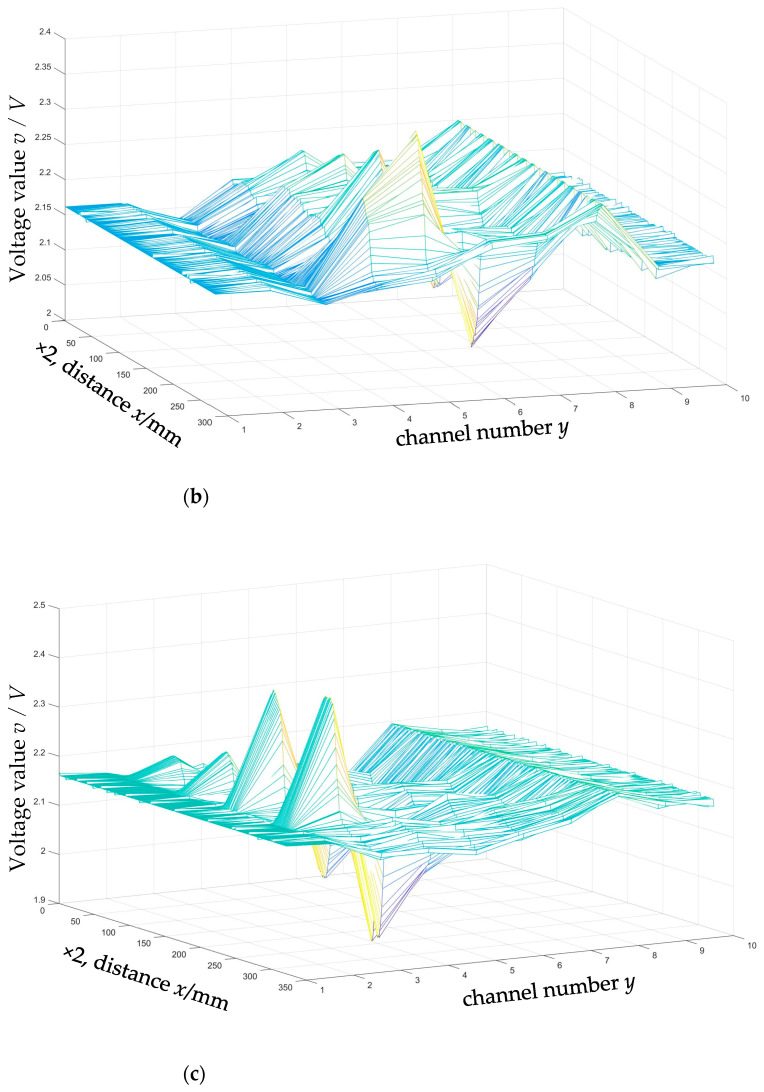
Magnetic flux leakage signal distribution of pipeline weld defects: (**a**) A defect-free X80 pipeline weld; (**b**) Cracks in the weld bead of the X80 pipeline; (**c**) Cracks in the fusion zone of the X80 pipeline weld.

Cracks in the middle of the weld and edge cracks in the weld of different sizes were prefabricated on the experimental pipeline weld. The schematic diagram is shown in Figure 7, and the parameters of the pipelines and cracks are shown in Table 1. The experimental process is shown in Figure 8. The magnetic flux leakage signal distribution of pipeline weld defects collected by the pipeline weld continuous non-contact magnetic flux leakage scanner developed with independent intellectual property rights is shown in Figure 9. The sampling system obtained the magnetic flux leakage signal data in Figure 9 by moving the detector to sample one data point per 2 mm. Therefore, the distance traveled by the detector = the number of sampling points × 2 mm.

Figure 9 illustrates that the magnetic flux leakage signal of the pipeline weld shows a downward and then upward trend, that is, the trough appears first and then the peak, showing a “trough-peak” trend. The magnetic flux leakage signal of the crack in the middle of the pipe weld shows a trend of first going up and then going down, that is, the peak appears first and then the trough appears. The superposition of the pipe weld signal and the crack signal shows a “trough-peak-trough-peak” trend. The edge cracks of the pipe weld show a trend of going up first and then going down, that is, the peak appears first and then the trough appears. Due to the location of the defect, the edge crack trough and the weld trough overlap, showing a “peak-trough-peak” trend. Through comparison with the numerical simulation analysis [26] related to this research group, it is consistent with the numerical simulation trend shown in the literature [26] when the weld has no defects and cracks exist at different positions of the weld. And as the depth size of the pipeline weld defect increases, the magnetic flux leakage signal shows an increasing trend, which is consistent with the numerical simulation trend of the weld crack expansion process shown in the literature [26].

## 5. Quantitative Analysis of Pipeline Weld Defects

As can be seen from Figure 9 in the previous section, the pipeline weld signal and the crack signal in the collected magnetic flux leakage signals overlap, which complicates the quantitative analysis of defects. Therefore, this paper uses the visualization method of image display to conduct the quantitative analysis. The grayscale linear transformation method is used to convert the collected magnetic flux leakage signals into digital images in the form of a grayscale matrix, and a quantitative research method for pipeline weld defects based on the fusion of mathematical morphology and chain code algorithm is proposed. With the help of the Delphi software visual programming tool, a pipeline weld defect inversion software system is developed, and the proposed method is implemented through the developed software system. The software not only has the functions of converting the collected magnetic flux leakage signals of pipeline weld defects into grayscale images and carrying out the mathematical morphology removal of small objects, binarization, and other mathematical morphology processing algorithms, but it also has the function of recording the chain code information of the defect boundary, so that characteristic parameters such as defect length and defect area can be calculated. According to the number of sensors designed by the continuous non-contact magnetic flux leakage scanning detector for pipeline welds, this software compiles 10 channel acquisitions.

**Figure 10:**
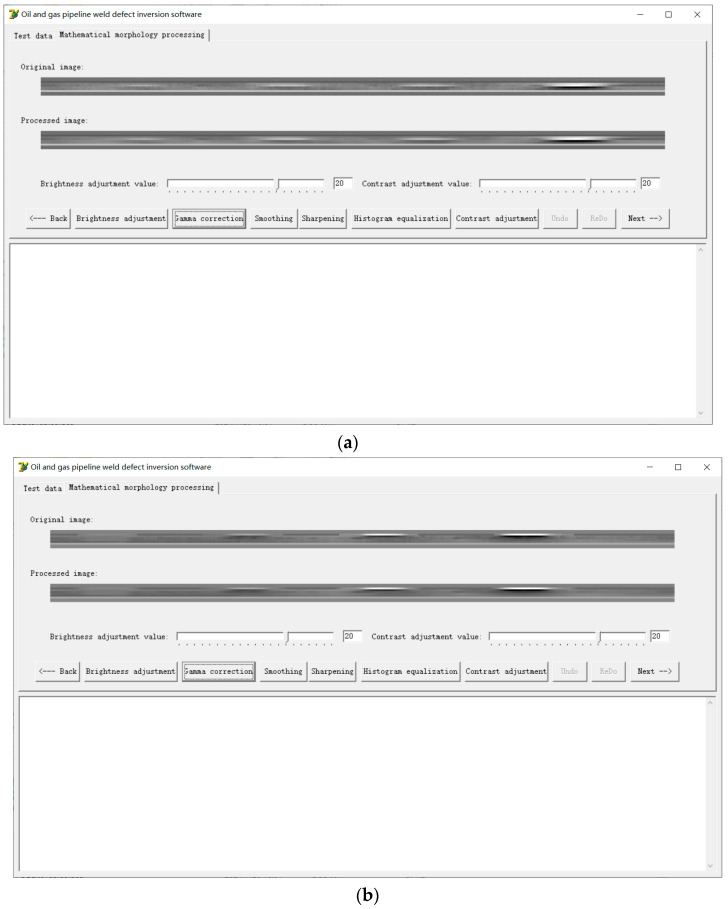
Grayscale image of pipeline weld defects: (**a**) Cracks in the weld bead of the X80 pipeline; (**b**) Cracks in the fusion zone of the X80 pipeline weld.

The collected magnetic flux leakage signal of pipeline weld defects is a matrix. The intersection of rows and columns is taken to mark each pixel of the image. Each pixel corresponds to a gray value. The collected magnetic flux leakage signals are converted into a grayscale image according to the grayscale linear transformation relationship. Different gray levels are used to represent the amplitude of the weld magnetic leakage signal, as shown in Figure 10. The grayscale plots in Figure 10a,b correspond to the magnetic flux leakage signal distribution curves in Figure 9b and Figure 9c, respectively. The abscissa value of the grayscale image pixel is the number of sampling points of the detection system; this multiplied by 2 mm is the travel distance. The ordinate value of the grayscale image pixel is determined by the number of channels in the sensor where the defective signal is located. Based on the grayscale image of the pipeline weld defects, corresponding mathematical morphology processing can be performed.

**Figure 11:**
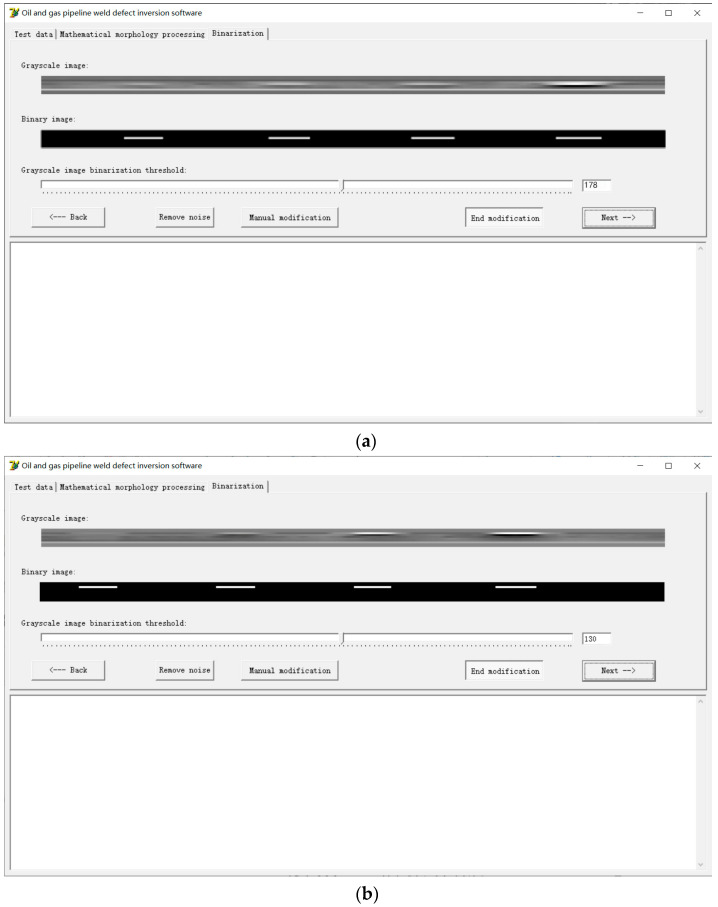
Binary segmentation: (**a**) Cracks in the weld bead of the X80 pipeline; (**b**) Cracks in the fusion zone of the X80 pipeline weld.

**Figure 12:**
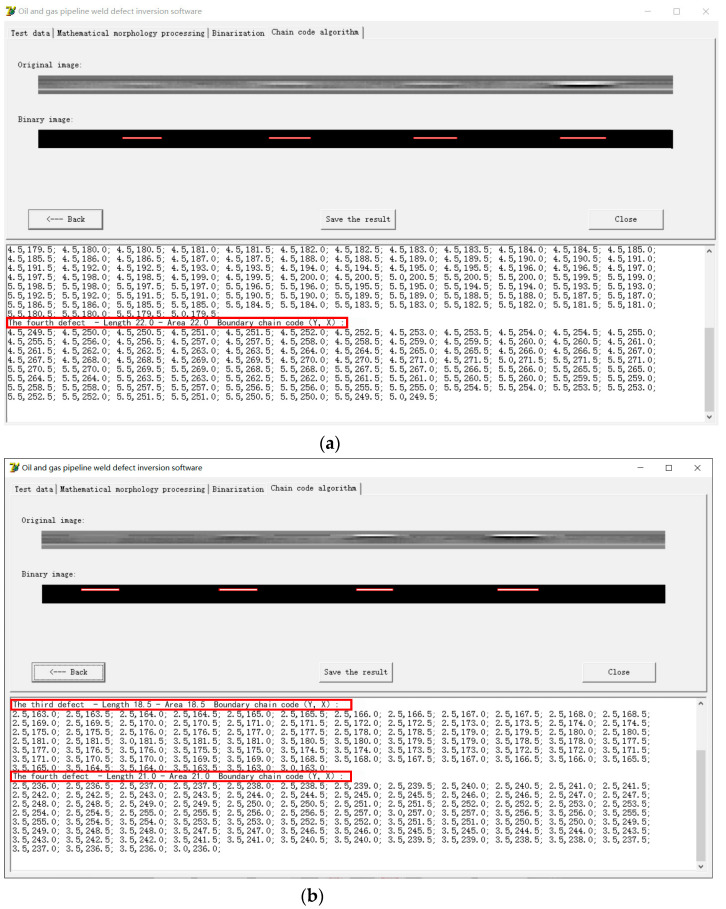
Chain code information of the defect boundary: (**a**) Cracks in the weld bead of the X80 pipeline; (**b**) Cracks in the fusion zone of the X80 pipeline weld.

The processed pipeline weld defect grayscale image is binarized and segmented using a binary algorithm, as shown in Figure 11. On this basis, the chain code information of the defect boundary is recorded using the compiled chain code algorithm, and the extraction of relevant feature quantities such as defect length and defect area is completed, as shown in Figure 12. All the defect widths are 1 mm, so after extracting the defect length, the defect area can be obtained. The defect length obtained from the chain code information shown in Figure 12 is shown in Table 2. Table 2 also gives the relative length error of each defect, where the relative error ∆r is calculated by the following formula:

(6) $$∆r=\frac{\left|Real\;defect\;length-Defect\;length\;displayed\;by\;chain\;code\right|}{Real\;defect\;length}\%$$

It is calculated from Table 2 that the maximum error of the measurement length of this method is 20%, and the minimum accuracy of the measurement length of this method is 80%. Thus, a preliminary quantitative study of pipeline weld defects has been achieved.

## 6. Discussion

(1) The traditional pipeline magnetic flux leakage detection device uses a detection system mode in which the magnetization direction is parallel to the direction of travel. However, due to the structural characteristics of the weld, the traditional detection system mode is not applicable. Since the weld magnetic flux leakage detection device needs to travel along the direction of the weld, the detector designed in this paper rotates the magnetizer 90 degrees along the direction of the weld seam so that the magnetization direction is perpendicular to the direction of travel, breaking through the technical barrier of traditional magnetic flux leakage detection devices not being suitable for weld detection.
(2) Currently, the device can detect pipeline weld defects such as cracks and corrosion, and the detection sensitivity can reach a 20% wall reduction. But it cannot detect rough surfaces, geometrical distortions, uneven weld beads, etc., in pipeline welds. For pipeline bend welds, since the detection device has a certain size, the detection device cannot pass through smaller bends, so it can only detect pipeline bend welds within a certain size range. In the future, consideration will be given to improving the detection device to break through the current limitations.
(3) The quantitative research method of pipeline weld defects proposed in this paper, which is based on the integration of mathematical morphology and the chain code algorithm, studies the quantification of crack defects from the geometric dimension changes in a single depth direction of the crack defects (dimensions in other directions remain unchanged), which has certain limitations. Future work will investigate methods to quantify the three-dimensional dimensions of defects.

## 7. Conclusions

(1) The magnetization direction of the detector designed in this paper is perpendicular to the direction of travel. Correspondingly, the sensor arrangement has also been changed. The sensors are arranged inside the sensor box parallel to the bottom surface of the pole shoes. The sensor array direction is parallel to the magnetization direction and perpendicular to the traveling direction. Through the above technological innovation, pipeline weld inspection based on the magnetic flux leakage method has been realized, broadening the scope of existing inspection objects.
(2) In order to match the contour of the weld, a sensor box that matches the reinforcement of the weld was designed. On one hand, it reduces the influence of fluctuations in the distance between the sensor and the pipe weld; that is, the lift-off value. On the other hand, it ensures that the lift-off value between the sensor and the pipe and the weld is equal, thereby improving the sensitivity of the detection signal.
(3) A quantitative research method for pipeline weld defects based on the integration of mathematical morphology and chain code algorithms was proposed, and a pipeline weld defect inversion software system was developed. Through the developed software, the mathematical morphological processing of magnetic flux leakage images of pipeline weld defects was realized, the chain code information of the defect boundary was extracted, and parameters such as defect length and defect area were measured. The minimum accuracy of measuring length by this method was 80%, and a preliminary quantitative study of pipeline weld defects was realized. This research content provides new ideas for quantitative research on pipeline weld defects.

## Figures and Tables

**Table 1:** Parameters of pipelines and cracks.

X80 Pipeline Parameter		
Pipeline diameter/mm		1219
Pipeline wall thickness/mm		18.4
Weld width/mm		22
Weld reinforcement/mm		2
Defect parameter		
Length 40 mm, Width 1 mm		
No.	Depth	Value/mm
1	Weld reinforcement + 20% of pipeline wall thickness	5.68
2	Weld reinforcement + 40% of pipeline wall thickness	9.36
3	Weld reinforcement + 60% of pipeline wall thickness	13.04
4	Weld reinforcement + 80% of pipeline wall thickness	16.72
5	20% of pipeline wall thickness	3.68
6	40% of pipeline wall thickness	7.36
7	60% of pipeline wall thickness	11.04
8	80% of pipeline wall thickness	14.72

**Table 2:** Relative error in the length measurements.

No.		Length/mm	No.		Length/mm
1	Real defect length/mm	40	5	Real defect length/mm	40
	Defect length displayed by chain code/mm	2 × 16		Defect length displayed by chain code/mm	2 × 18
	Relative error ∆r/%	20		Relative error ∆r/%	10
2	Real defect length/mm	40	6	Real defect length/mm	40
	Defect length displayed by chain code/mm	2 × 19		Defect length displayed by chain code/mm	2 × 18
	Relative error ∆r/%	5		Relative error ∆r/%	10
3	Real defect length/mm	40	7	Real defect length/mm	40
	Defect length displayed by chain code/mm	2 × 21		Defect length displayed by chain code/mm	2 × 18.5
	Relative error ∆r/%	5		Relative error ∆r/%	7.5
4	Real defect length/mm	40	8	Real defect length/mm	40
	Defect length displayed by chain code/mm	2 × 22		Defect length displayed by chain code/mm	2 × 21
	Relative error ∆r/%	10		Relative error ∆r/%	5

## Data Availability

The original contributions presented in the study are included in the article, further inquiries can be directed to the corresponding authors.
